# CRISPR/Cas9-generated models uncover therapeutic vulnerabilities of del(11q) CLL cells to dual BCR and PARP inhibition

**DOI:** 10.1038/s41375-020-0714-3

**Published:** 2020-01-23

**Authors:** Miguel Quijada-Álamo, María Hernández-Sánchez, Verónica Alonso-Pérez, Ana E. Rodríguez-Vicente, Ignacio García-Tuñón, Marta Martín-Izquierdo, Jesús María Hernández-Sánchez, Ana B. Herrero, José María Bastida, Laura San Segundo, Michaela Gruber, Juan Luis García, Shanye Yin, Elisa ten Hacken, Rocío Benito, José Luis Ordóñez, Catherine J. Wu, Jesús María Hernández-Rivas

**Affiliations:** 1 University of Salamanca, IBSAL, IBMCC, CSIC, Cancer Research Center, Salamanca, Spain; 2 Department of Hematology, University Hospital of Salamanca, Salamanca, Spain; 3 Department of Medical Oncology, Dana-Farber Cancer Institute, Boston, MA 02115 USA; 4 Broad Institute of Harvard and MIT, Cambridge, MA 02142 USA; 5 CeMM Research Center for Molecular Medicine, Vienna, Austria; 6 Department of Internal Medicine I, Division of Hematology and Hemostaseology, Medical University of Vienna, Vienna, Austria; 7 Department of Medicine, University of Salamanca, Salamanca, Spain

**Keywords:** Chronic lymphocytic leukaemia, Cytogenetics

## Abstract

The deletion of 11q (del(11q)) invariably comprises *ATM* gene in chronic lymphocytic leukemia (CLL). Concomitant mutations in this gene in the remaining allele have been identified in 1/3 of CLL cases harboring del(11q), being the biallelic loss of *ATM* associated with adverse prognosis. Although the introduction of targeted BCR inhibition has significantly favored the outcomes of del(11q) patients, responses of patients harboring ATM functional loss through biallelic inactivation are unexplored, and the development of resistances to targeted therapies have been increasingly reported, urging the need to explore novel therapeutic approaches. Here, we generated isogenic CLL cell lines harboring del(11q) and *ATM* mutations through CRISPR/Cas9-based gene-editing. With these models, we uncovered a novel therapeutic vulnerability of del(11q)/*ATM*-mutated cells to dual BCR and PARP inhibition. Ex vivo studies in the presence of stromal stimulation on 38 CLL primary samples confirmed a synergistic action of the combination of olaparib and ibrutinib in del(11q)/*ATM*-mutated CLL patients. In addition, we showed that ibrutinib produced a homologous recombination repair impairment through RAD51 dysregulation, finding a synergistic link of both drugs in the DNA damage repair pathway. Our data provide a preclinical rationale for the use of this combination in CLL patients with this high-risk cytogenetic abnormality.

## Introduction

Deletion of chromosome 11q22.3 (del(11q)) can be found in up to 20% of chronic lymphocytic leukemia (CLL) patients at diagnosis and is associated with poor outcome [1–3]. Although the size of this deletion is variable [4–6], *ATM* is consistently deleted in most cases [6–8]. This gene, which plays a central role in double-strand break (DSB) signaling and repair [9], is mutated in 10–20% of CLL cases at diagnosis [10–13]. One-third of CLL patients with del(11q) carry *ATM* mutations in the remaining allele, resulting in complete loss-of-function of the ATM protein [14] and significantly reducing the survival of these patients [15].

Novel agents targeting BCL2 and BCR signaling pathways have revolutionized the treatment landscape in CLL [16]. Specifically, it has been recently reported that treatment-naïve del(11q) CLL patients show durable responses upon first-line ibrutinib treatment [17] and an integrated analysis of long-term follow-up data from three randomized trials of ibrutinib in CLL revealed that ibrutinib-treated patients with del(11q) had a significantly longer progression-free survival than ibrutinib-treated patients without del(11q) [18]. However, responses to ibrutinib of high-risk patients harboring ATM functional loss through biallelic inactivation have not been explored yet. In addition, survival outcomes are inferior for relapsed/refractory CLL patients, including those with del(11q) [19], and resistance to BTK inhibitors is becoming an increasing therapeutic challenge [20–24]. For these reasons, novel combinatorial therapies need to be explored in CLL patients.

One of the major impediments to the study of CLL biology has been the lack of cellular models faithfully representing the key genetic events of this disease, such as del(11q). While some studies have interrogated the biological impact of diverse individual CLL-associated genetic alterations [25–29], very few have analyzed the effects of concurrently expressed mutations in CLL [30]. Recently, Clustered Regularly Interspaced Short Palindromic Repeats (CRISPR)/Cas9 technology has allowed the efficient generation of mutations and chromosomal alterations in human cell lines and animal models, opening new approaches for modeling human diseases [31–34]. These new capabilities provide fresh opportunities to generate cell lines to mimic the concurrence of genetic alterations and to study specific therapeutic options.

In the present study, we used the CRISPR/Cas9 technology to generate stable isogenic CLL-derived cell lines harboring del(11q) and/or *ATM* mutations. The loss of *ATM* by del(11q) and gene mutation led to a defective double-strand break (DSB) signaling resulting in increased genomic instability and hypersensitivity to the PARP inhibitor olaparib in vitro*,* in vivo and ex vivo. Furthermore, we showed that ibrutinib synergizes with PARP inhibition triggering synthetic lethality and significantly improving the effects of BCR inhibition as monotherapy in del(11q) cell lines and primary CLL cells. In addition, we demonstrated that the synergy mechanism between both is associated with the effect of ibrutinib in interfering with the homologous recombination repair through RAD51 downregulation. Our studies suggest that CRISPR/Cas9-generated models may provide powerful tools to study the effects of individual or combined CLL genetic alterations on cellular processes and treatment response.

## Methods

### Study approval

The ex vivo study was conducted in accordance of the Declaration of Helsinki and prior approval by the Bioethics Committee from our institution. Written informed consent was obtained from all patients. Animal studies were conducted in accordance with the Spanish and European Union guidelines for animal experimentation (RD53/2013, Directive-2010/63/UE, respectively) and received prior approval from the Bioethics Committee of our institution.

### Primary CLL samples

Peripheral blood mononuclear cells (PBMCs) from 38 CLL patients were isolated using Ficoll-Paque Plus density gradient media (GE Healthcare, Life Sciences) and viably cryopreserved in liquid nitrogen until the time of analysis. A complete immunophenotypic analysis of all cases was carried out by flow cytometry. The main biological features of the CLL patients used in the study are summarized in Supplementary Table S1. Only CLL samples with CD19+/CD5+ purities greater than 85% were included.

### Next-generation sequencing (NGS)

NGS results from the primary samples used in the ex vivo experiments are detailed in Supplementary Tables S2 and S3. Full details in Supplementary Information.

### CRISPR/Cas9-mediated mutagenesis in CLL cell lines

HG3 and MEC1 cell lines (which harbor del(13q) and del(17p), respectively) were transduced with lentiviral particles containing plasmids for the constitutive Cas9 expression (LentiCas9-Blast, Addgene_#52692).

SgRNAs were designed using the online CRISPR design tool (http://crispr.mit.edu/) to target *ATM*. The selection of the sgRNAs was based on choosing those of highest efficiency to target the gene of interest and with the lowest predicted off-targets effects. For the generation of del(11q) on the HG3 cell line, two sgRNAs were designed targeting two ∼17 Mb distal regions on chromosome 11 (11q22.1 and 11q23.3, respectively). In addition, a sgRNA designed not to target the human genome was used as a negative control. Sequences of the selected sgRNAs are detailed in Supplementary Table S4. SgRNAs targeting 11q23.3 were cloned into pLKO5.sgRNA.EFS.GFP (Addgene_#57822) and sgRNAs targeting *ATM* and 11q22.1 into pLKO5.sgRNA.EFS.tRFP (Addgene_#57823). Negative control sgRNA was cloned in both vectors. Cloning was carried out as previously described [35] and lentiviral transduction, nucleofection of 11q-targeting sgRNAs and clone screening are detailed below. At least three different clones harboring loss-of-function mutations were chosen for each CRISPR-generated cell line to perform further functional studies. To mitigate against the possible biases due to off-targets effects of the sgRNAs, clones were generated using two different sgRNAs per gene.

### Ex vivo experiments

Primary CLL ex vivo experiments were carried out in the presence of HS-5 bone marrow stromal cells as previously described [36]. Briefly, HS-5 stromal cells were seeded 24 h prior to the ex vivo study at a concentration of 1.5 × 10^4^ cells/mL. On the following day, primary CLL cells were viably unfrozen and resuspended in RPMI 1640 medium (Life Technologies) supplemented with 10% FBS, 1% penicillin/streptomycin and 1.5 μg/mL CpG ODN (Sigma-Aldrich) plus 50 ng/mL IL-2 (Peprotech) and subsequently seeded onto the HS-5 cell layer at a co-culture ratio of 100:1 (1.5 × 10^6^ CLL cells /mL) to stimulate proliferation of CLL cells [37].

Cells were γ-irradiated (2 Gy) 24 h after co-culture for γH2AX experiments. In the cell viability experiments, CLL cells in the co-culture condition were treated with the indicated drug doses on each experiment. After 120 h, CLL cells were detached from the stromal cell layer and viability was measured by CellTiter-Glo Luminescent Assay (Promega) and normalized with cells with no drug treatment.

### Statistics

Statistical significance was determined using GraphPad Prism software v6 (GraphPad Software). Data are summarized as the mean ± standard deviation (SD). Otherwise specified three independent clones per condition were used in the functional studies. Student’s *t* test, Mann–Whitney, ANOVA or Kruskal–Wallis tests were used to determine statistical significance. *P* values lower than 0.05 were considered as statistically significant.

### Supplemental methods

Supplemental Methods section include detailed protocols of cell lines and culture conditions, NGS, lentiviral production, cell transduction and nucleofection, PCR, FISH, flow cytometry, western blot, viability and apoptosis analyses, immunofluorescence, comet assay, transwell migration assay, homologous recombination (HR) activity assay and in vivo experiments.

## Results

### Generation of del(11q) and *ATM*-deficient isogenic CLL-derived cell lines using the CRISPR/Cas9 system

To address the biological implications of del(11q) and/or *ATM* mutations in CLL, HG3 and MEC1 CLL-derived cell lines were selected. Both are diploid for chromosome 11 and have wild-type (WT) *ATM* gene. Both cell lines were transduced with lentivirus expressing a constitutive Cas9 protein, generating HG3-Cas9 and MEC1-Cas9 cell lines with a Cas9 activity greater than 80% (Supplementary Fig. S1a, b).

**Fig. 1:**
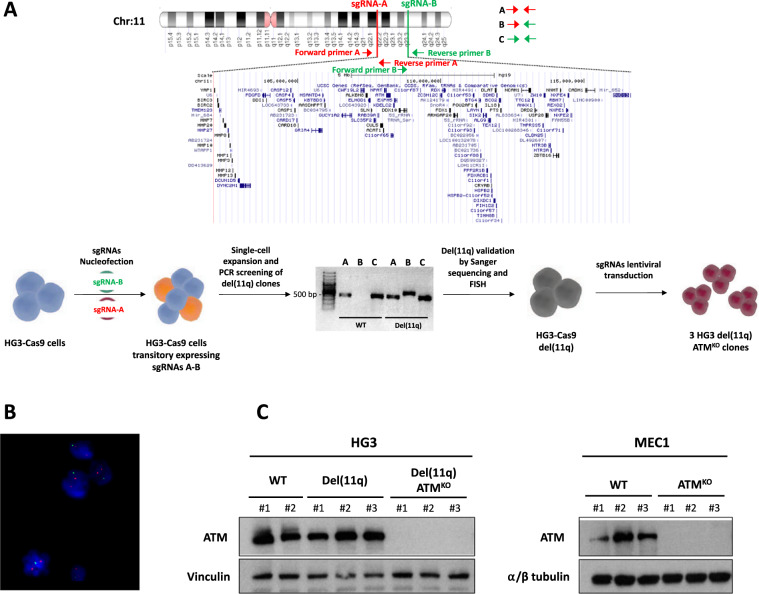
Generation of 11q deletion and *ATM* mutations in CLL cell lines using the CRISPR/Cas9 system. **a** Upper panel presents the design of the generation of 11q deletion in HG3 cells and the genes contained within the two sgRNA-targeting sites (sgRNA-A in red, sgRNA-B in green). PCR primers used for detection of the deletion are indicated by arrows. Lower panel shows a diagram with the steps for the generation of an HG3-del(11q) cell line. Single-cell sorted clones transitory expressing sgRNAs A and B were screened for the presence of del(11q) by PCR reactions A, B and C, using two pairs of primers flanking upstream and downstream sgRNAs cut sites on chromosome 11. Only del(11q)-positive clones showed amplification using the forward primer A (11q22.1) and the reverse primer B (11q23.3) (indicated as “PCR B”), as a result of a fusion product between both cut sites A and B. HG3-del(11q) isogenic cell line was subsequently used for the generation of *ATM* mutations on the remaining wild-type allele of HG3-del(11q) cells. In total, *n* = 3 HG3-del(11q) *ATM*^KO^ clones were generated. **b** FISH analysis of HG3-del(11q) cell line. Green signals correspond to 11q22/*ATM* probe and the control red signals correspond to 17p13/*TP53* probe. **c** Western blot analyses of HG3-del(11q) and MEC1 edited single-cell clones with *ATM* mutations (*n* = 3 clones per condition).

For the generation of del(11q), HG3-Cas9 cells were nucleofected with two sgRNAs targeting specific regions on chromosome 11q22.1 (sgRNA-A) and 11q23.3 (sgRNA-B), respectively. After single-cell sorting of GFP + RFP + cells, clones were screened by PCR for the presence of a fusion region between 11q22.1 and 11q23.3 (Fig. 1a). Monoallelic del(11q) was present in 100% of the cells of the selected clone as validated by FISH (Fig. 1b), thereby establishing an isogenic HG3-del(11q) cell line. Truncating mutations of *ATM* were introduced on the remaining WT allele of HG3-del(11q) cells (Fig. 1a). Single-cell FACS-sorted clones were sequenced and the absence of ATM functional protein was assessed by western blot (Fig. 1c). In total, we generated three different clones of HG3-del(11q) and HG3-del(11q) *ATM*^KO^ conditions. A similar approach was used to generate single-cell clones with single *ATM* mutations in MEC1 and HG3 cell lines, validating the loss of ATM in three clones per condition (Fig. 1c; Supplementary Fig. S2a, b).

### Del(11q) *ATM*^KO^ cells show impaired double-strand breaks signaling, leading to DNA damage accumulation

**Fig. 2:**
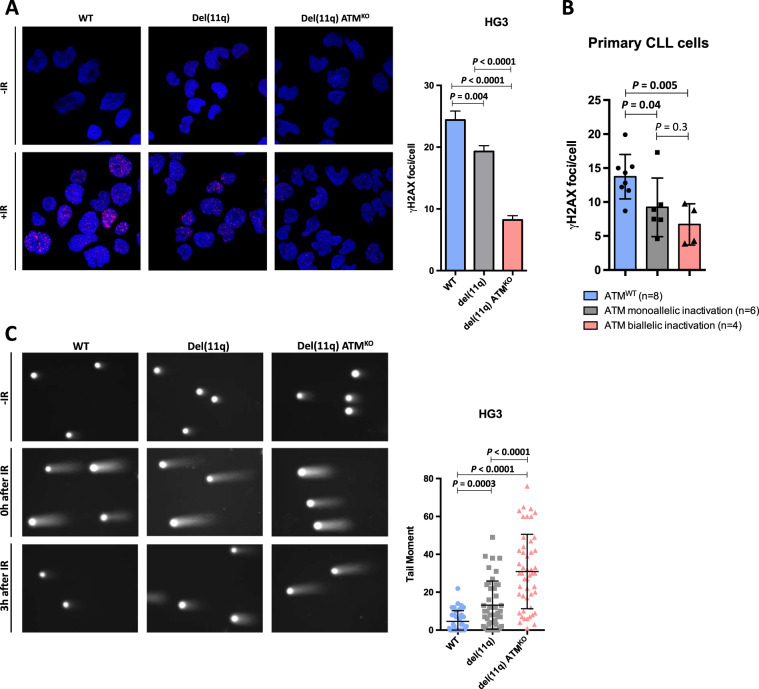
Evaluation of double-strand breaks signaling and repair in del(11q)/*ATM*-deficient CLL cells. **a** Left panel: representative images of γH2AX foci formation (red) in HG3^WT^, HG3-del(11q) and HG3-del(11q) *ATM*^KO^ clones. Upper panel shows non-irradiated (−IR) HG3 cells and lower panel represents HG3 clones 1 h after 2 Gy irradiation (+IR). Right panel: quantification of the number of γH2AX foci per cell 1 h after irradiation. Data are represented as the mean values ± SD of three independent experiments. At least 75 cells per experiment were counted. **b** Quantification of the number of γH2AX foci per cell 1 h after irradiation in primary CLL samples stimulated to proliferate for 24 h before IR (2 Gy). Groups are stratified based on *ATM*^WT^ (*n* = 8) *ATM* monoallelic (*n* = 6) or biallelic (*n* = 4) defects in CLL samples. At least 75 cells per patient were counted. Primary samples used in this experiment are detailed in Supplementary Table 1**. c** Left panel: representative images of the neutral comet assay experiment in HG3^WT^, HG3-del(11q) and HG3-del(11q) *ATM*^KO^ clones. Upper images show non-irradiated HG3 comets, middle panel represents comets right after 40 Gy irradiation and lower images present comets 3 h after 40 Gy irradiation, when HG3^WT^ were able to repair the IR-generated DNA damage. Right panel: tail moment quantification of neutral comet assays in HG3^WT^, HG3-del(11q) and HG3-del(11q) *ATM*^KO^ clones 3 h after 40 Gy irradiation. Data represent the mean values ± SD of at least 50 comets analyzed per condition in three independent experiments.

ATM is known to phosphorylate histone H2AX in response to DSBs, promoting DSB repair [9]. To test how the CRISPR/Cas9-engineered CLL cells respond to DSBs, γH2AX foci formation was investigated in the presence or absence of exposure to γ-irradiation (IR). By immunofluorescence, the number of foci was markedly lower in HG3-del(11q) clones than in HG3^WT^ cells following IR exposure (*P* = 0.004; Fig. 2a). In addition, biallelic inactivation of *ATM* dramatically reduced the number of foci formed after IR (Fig. 2a). These results were corroborated in HG3 and MEC1 *ATM*^KO^ cells (Supplementary Fig. S3a, b) as well as in del(11q)/*ATM*-mutated primary CLL cells (Fig. 2b).

Since del(11q) cells displayed impaired DNA damage signaling, neutral comet assays were performed to elucidate whether these cells presented DSB accumulation after γ-irradiation. Notably, all the HG3 clones harboring del(11q) showed DNA damage accumulation 3 h after IR whereas HG3^WT^ cells were able to repair the DSBs (Fig. 2c). Furthermore, comet analyses revealed that the tail moment was higher in HG3 cells with biallelic inactivation of *ATM* than HG3-del(11q) cells with the remaining *ATM*^WT^ allele (*P* < 0.0001; Fig. 2c).

### CRISPR/Cas9-engineered del(11q)/*ATM*^KO^ CLL cells show high sensitivity to PARP inhibition in vitro and in vivo

**Fig. 3:**
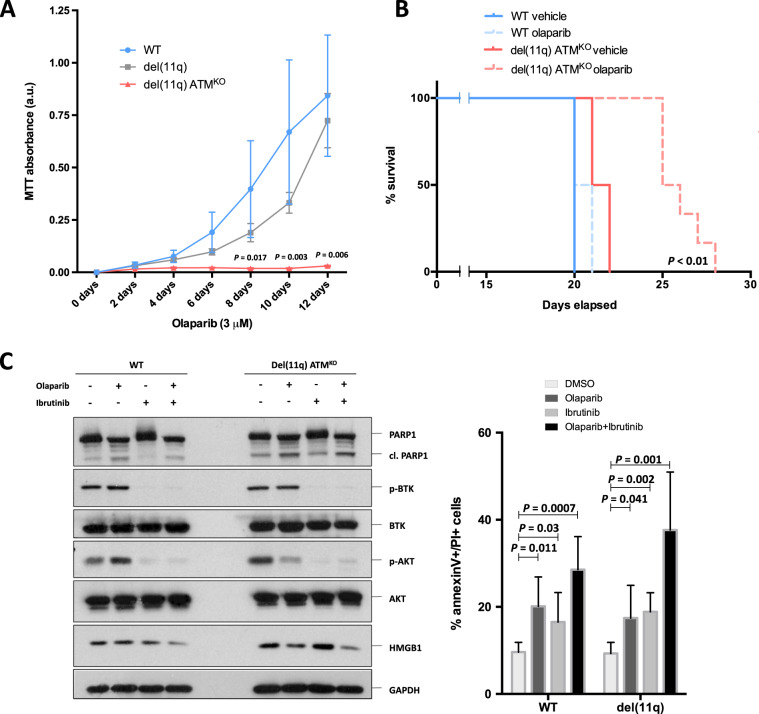
Olaparib effects in CRISPR/Cas9-edited del(11q) cells in vitro, in vivo and in combination with ibrutinib. **a** HG3-edited clones were treated with 3 μM olaparib and cell viability was assessed by MTT every 2 days up to 12 days. Proliferation rates are presented as MTT absorbance units, and data are shown as mean ± SD. *P* values indicate differences between HG3^WT^ and HG3-del(11q) *ATM*^KO^ clones. **b** Kaplan–Meier overall survival curve of HG3^WT^ (*n* = 10) and HG3-del(11q) *ATM*^KO^ (*n* = 10) xenografted mice treated with olaparib (*n* = 6) or vehicle (*n* = 4). The reported *P* value was calculated by Log-rank test. **c** Left panel: immunoblot analysis of whole-cell lysates of HG3^WT^ and HG3-del(11q) *ATM*^KO^ cells exposed to 5 μM olaparib and ibrutinib, either alone or in combination, after 48 h. Right panel: cytotoxicity studies by annexin V/PI staining of HG3^WT^ and HG3-del(11q) cells treated with 5 μM olaparib and ibrutinib for 48 h. Cytotoxicity is measured as the percentage of PI + and annexin V + cells. Data are summarized as the mean ± SD of four independent experiments.

Considering that del(11q) and *ATM* mutations lead to defective DNA repair, we hypothesized that these cells could be hypersensitive to other drugs that also interfere the DNA repair pathways, such us PARP inhibitors [38]. To analyze this, HG3 clones were treated with olaparib. Of note, clones with biallelic inactivation of *ATM* due to del(11q) and mutation on the other allele showed incipient sensitivity 72 h after treatment (Supplementary Fig. S4a). In addition, proliferation assays confirmed that del(11q) *ATM*^KO^ cells could not proliferate after olaparib treatment even after prolonged exposure (Fig. 3a). These results were also confirmed in MEC1 *ATM*^KO^ clones (Supplementary Fig. S4b).

To investigate the in vivo impact of olaparib treatment, HG3^WT^ (*n* *=* 10) and HG3-del(11q) *ATM*^KO^ (*n* = 10) xenografts were generated in NSG mice. Olaparib or vehicle treatment started one week after cell injection (vehicle *n* *=* 4; olaparib *n* *=* 6) and hCD45 + GFP + populations in the peripheral blood were monitored twice weekly. By FACS analysis, slower leukemic progression was observed in the del(11q) *ATM*^KO^ mice treated with olaparib (*P* = 0.004, at day 16 post-injection) whereas no differences were found between vehicle or olaparib treated WT xenografts (Supplementary Fig. S4c). Finally, overall survival was assessed at the end of the experiment, showing a significantly longer survival of del(11q) *ATM*^KO^ xenografts treated with olaparib as compared with control (*P* < 0.01) (Fig. 3b).

### Ibrutinib has a synergistic effect with olaparib in vitro, enhancing its cytotoxic effects in del(11q) CLL cells

To test whether the CRISPR/Cas9-engineered CLL cell lines could be used as models to pre-clinically test new therapeutic approaches, and given the promising effects of olaparib on del(11q)/*ATM*-mutated CLL cells, synergy experiments were performed to test whether PARP inhibition could be combined with other drugs employed in CLL therapy. Strikingly, BCR inhibition by ibrutinib potentiated the effects of olaparib in cell viability in all the HG3-del(11q) and HG3-del(11q) *ATM*^KO^ CRISPR/Cas9-generated clones and MEC1 cells (Supplementary Fig. S5a, b). Furthermore, olaparib also synergized with the alkylating agent bendamustine in all HG3-edited cell lines (Supplementary Fig. S5a). Responses of these isogenic HG3 cells to ibrutinib and bendamustine in monotherapy are depicted in Supplementary Fig. S5c.

We next focused on the combination of olaparib and ibrutinib due to its potential therapeutic application in del(11q)/*ATM*-mutated-relapsed/refractory CLL patients. As expected, the combination of these drugs induced PARP cleavage added to p-BTK and downstream p-AKT inhibition (Fig. 3c, left panel). In addition, ibrutinib synergistically enhanced olaparib cytotoxicity, leading to an incremented cell death, mostly by necrosis, as shown by annexin V and PI staining (Fig. 3c, right panel; Supplementary Fig. S5d, e). Moreover, necrosis-dependent HMGB1 release [39] was studied in response to the drugs alone or in combination, finding a marked reduction of HMGB1 levels in HG3^WT^ and HG3-del(11q) *ATM*^KO^ cells exposed to the combination of olaparib and ibrutinib (Fig. 3c). Furthermore, we tested whether this combination affected the CCL19-mediated migration by chemotaxis assays, revealing that the drug combination at non-cytotoxic doses significantly reduced migration of CLL cell lines towards CCL19 (Supplementary Fig. S5f).

### Dual BCR and PARP inhibition is highly effective in del(11q)/*ATM*-mutated primary CLL samples in the presence of stromal stimulation

**Fig. 4:**
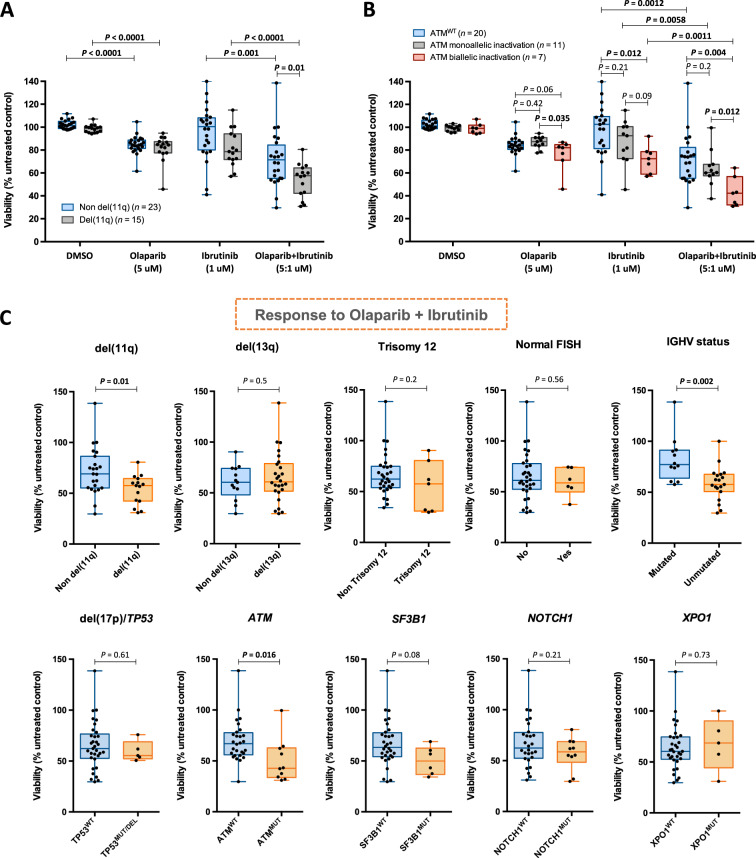
Response to dual BCR and PARP inhibition of 38 CLL primary samples in the presence of stromal stimulation. **a, b** Primary CLL cells were seeded in co-culture with HS-5 bone marrow stromal cells, 1.5 μg/mL CpG and 50 ng/mL IL-2 and treated with olaparib (5 μM), ibrutinib (1 μM) or the combination of both for 5 days. Normalized surviving fraction is expressed relative to untreated cells. Data are presented as the mean ± SD of del(11q) (*n* *=* 15) vs. non del(11q) (*n* *=* 23) (**a**) or *ATM*^WT^ (*n* *=* 20); *ATM* monoallelic defective (*n* *=* 11) and *ATM* biallelic defective (*n* *=* 7) CLL cases **(b)**. **c** Response of primary CLL cells (*n* = 38) to the combination of olaparib (5 μM) and ibrutinib (1 μM) based on cytogenetics, IGHV mutational status and mutations in known CLL driver genes. Cells were seeded in co-culture with HS-5 bone marrow stromal cells, 1.5 μg/mL CpG and 50 ng/mL IL-2 and treated with the drug combination for 5 days.

In order to validate whether these del(11q)/*ATM*-mutated CRISPR/Cas9 models could be used as a predictive preclinical tool for the study of novel therapeutic approaches, we examined the effects of the combination of olaparib and ibrutinib ex vivo in primary cells from a cohort of 38 CLL samples (non-del(11q) *n* *=* 23; del(11q) *n* *=* 15). Given that olaparib exerts its action during G2/M cell cycle phase, CLL primary cells were stimulated to proliferate in the presence of stromal cells, CpG and IL-2 [37]. Consistently, the combination of olaparib and ibrutinib in stimulated primary CLL cells was synergistic, and more effective in those cases with del(11q) (Fig. 4a). The tested drug doses did not affect the viability of the HS-5 stromal cells used in the co-culture with CLL cells (Supplemental Fig. S6a). Furthermore, stratifying these samples by *ATM* monoallelic or biallelic inactivation, we observed that CLL cells harboring *ATM* biallelic inactivation were even more sensitive to dual BCR and PARP inhibition (Fig. 4b).

Next, we sought to determine if other traditional prognostic factors would predict response to the combination of BCR and PARP inhibition. Regarding FISH cytogenetic alterations, we found that only del(11q) patients, and not del(13q), trisomy 12 or normal karyotype patients, had significantly higher ex vivo sensitivity to the drug combination (Fig. 4c). In addition, by stratifying the cohort regarding IGHV mutational status, we detected an improved response of IGHV unmutated patients when compared with those IGHV mutated (Fig. 4c), in line with previous results showing a higher sensitivity of unmutated CLLs to ibrutinib [40]. In order to evaluate which genetic maker could have a greater influence on drug response, we compared IGHV unmuted/*ATM*^WT^ group versus cases with *ATM* biallelic inactivation, showing that CLL patients with biallelic loss of *ATM* were more sensitive to the drug combination (Supplemental Fig. S6b). In addition, we dissected the response to dual BCR and PARP inhibition based on the mutational status of known CLL driver genes, demonstrating that only patients harboring *ATM* mutations significantly correlated with a greater sensitivity to the drug combination (Fig. 4c). Interestingly, *SF3B1* mutations, which also play a role on the DNA damage response [30, 41], showed a trend towards higher sensitivity to the combination as well. On the other hand, *TP53*, *NOTCH1* or *XPO1* mutations did not have an influence on the response to the combination of olaparib and ibrutinib (Fig. 4c).

### BCR inhibition impairs homologous recombination repair through RAD51 downregulation

**Fig. 5:**
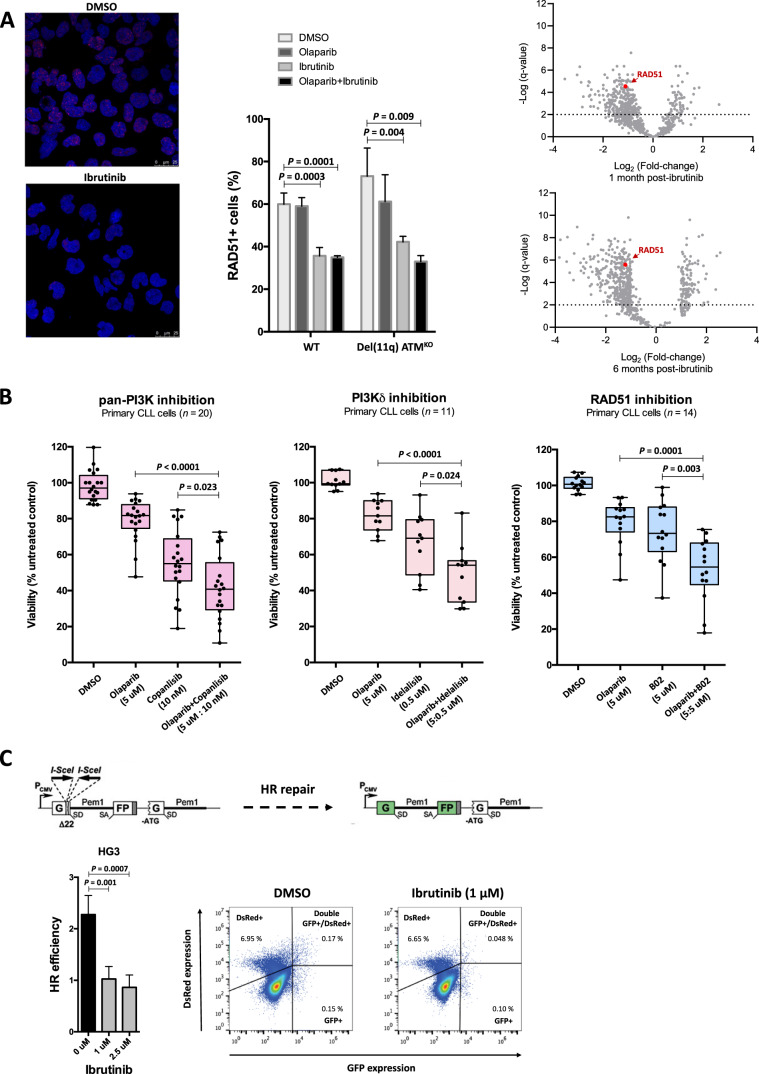
Effects of ibrutinib in RAD51-mediated HR repair in CLL. **a** Left panel: representative images and quantification of the number RAD51-positive cells 6 h after irradiation (2 Gy) in HG3^WT^ and HG3-del(11q) *ATM*^KO^ clones. Cells were pretreated for 24 h with 5 μM olaparib, 1 μM ibrutinib or the drug combination. Data are represented as the mean values ± SD of three independent experiments. Cells were scored RAD51 + when five or more foci were formed. At least 100 cells per experiment were counted. Right panel: volcano plots of transcripts changes comparing 1- (top) and 6-month (bottom) post-ibrutinib initiation vs. pretreated longitudinal samples in 14 CLL patients. *RAD51* expression is significantly downregulated in samples after 1 month and 6 months of ibrutinib therapy. Log_2_ of fold-changes (treatment vs. control) are shown in *x* axis and statistical significance (-log_10_ of *q* value) is shown in *y* axis. RNA-seq data were previously generated in Landau et al. [23]. **b** Primary CLL cells were seeded in co-culture with HS-5 bone marrow stromal cells, 1.5 μg/mL CpG and 50 ng/mL IL-2 and treated with the indicated drugs and doses for 5 days. Normalized surviving fraction is expressed relative to untreated cells. Data are presented as the mean ± SD. **c** Upper panel displays a representation of the HR-reporter plasmid adapted from Seluanov et al. [43]. Lower-left panel represents the HR repair efficiency as calculated by dividing the number of GFP + cells of the totality of positive-transfected DsRed + cells. Data represent mean ± SD of three independent experiments. Right panel displays representative plots of the HR efficiency of HG3 treated with DMSO or ibrutinib (1 μM).

Considering that olaparib and ibrutinib exert its action through different pathways, we hypothesized that the synergistic effects of this combination could be due to an off-target effect of ibrutinib in DNA damage repair. To determine if BTK inhibition affected the assembly of DNA repair foci on CLL cells, we investigated whether HG3^WT^ and HG3-del(11q) *ATM*^KO^ cells were able to recruit RAD51 to DSBs after γ-irradiation in the presence of ibrutinib. Surprisingly, the formation of RAD51 foci 6 h after IR was significantly reduced in CLL cells treated with ibrutinib or the combination than in untreated cells (Fig. 5a, left panel). In addition, ibrutinib treatment reduced the protein levels of RAD51 in these clones (Supplementary Fig. S7a). Similar results were obtained in MEC1 cells treated with ibrutinib (Supplementary Fig. S7b). We further investigated these findings through the analysis of transcriptomic RNA-seq data of serial samples of CLL patients treated with ibrutinib [23], confirming that RAD51 RNA levels are significantly reduced in CLL patients after 1 month and 6 months of ibrutinib therapy (Fig. 5a, right panel).

Moreover, we determined whether this RAD51 downregulation after ibrutinib exposure could be related to the downstream PI3K/AKT/mTOR signaling modulation of BTK inhibition. To this extent, we first used the pan-specific PI3K inhibitior copanlisib in the CRISPR/Cas9-generated cells, showing that PI3K inhibition also reduced the recruitment of RAD51 to DSB lesions (Supplementary Fig. S7c), presenting synergistic effects with olaparib in stimulated primary CLL cells (Fig. 5b, left panel) and CLL cell lines (Supplementary Fig. S7d). In addition, the selective PI3Kδ inhibitor idelalisib also showed synergism with olaparib in primary CLL cells (Fig. 5b, middle panel). In line with these results, the RAD51 inhibitor B02 also synergized with olaparib in stimulated CLL primary samples (Fig. 5b, right panel) and HG3^WT^ and HG3-del(11q) *ATM*^KO^ CRISPR/Cas9-edited cells (Supplementary Fig. S7d).

We next examined whether ibrutinib-mediated downregulation of RAD51 could reduce the homologous recombination (HR) repair activity of HG3 cells. Thus, we used an HR-reporter plasmid [42, 43] where GFP expression is restored upon HR repair (Fig. 5c). Consistently, ibrutinib treatment reduced the HR repair activity of HG3 cells at 1 and 2.5 μM concentrations (Fig. 5c).

### Ibrutinib enhances olaparib and bendamustine-dependent accumulation of DSBs in del(11q) cells

**Fig. 6:**
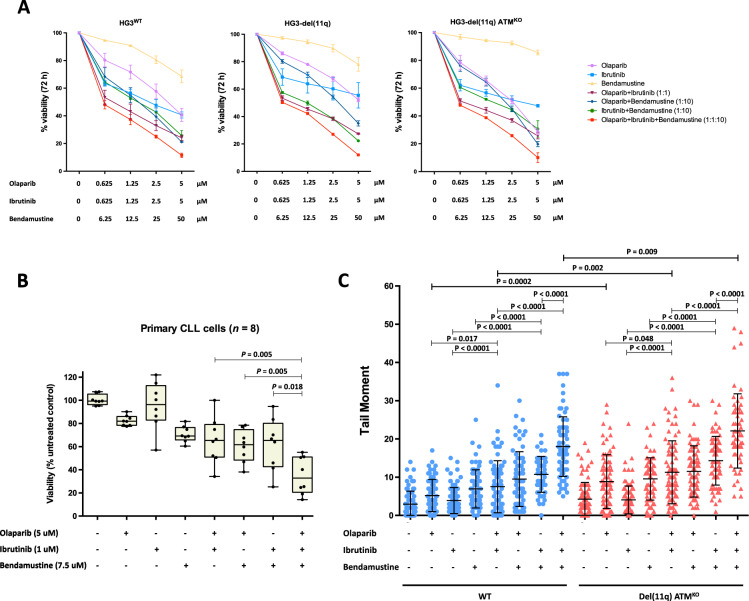
Implications of BCR inhibition in HR-mediated DSBs repair in CRISPR/Cas9-edited clones and primary CLLs. **a** HG3^WT^, HG3-del(11q) and HG3-del(11q) *ATM*^KO^ cells were treated with olaparib, ibrutinib and/or bendamustine and cell viability was assessed by MTT assay 72 h later. Surviving fraction is expressed relative to untreated controls and data are presented as the mean ± SD of two independent experiments. **b** Primary CLL cells were seeded in co-culture with HS-5 bone marrow stromal cells, 1.5 μg/mL CpG and 50 ng/mL IL-2 and treated with the indicated doses of olaparib, ibrutinib and/or bendamustine for 5 days. Normalized surviving fraction is expressed relative to untreated cells. **c** Tail moment quantification of neutral comet assays in HG3^WT^ (blue) and HG3-del(11q) *ATM*^KO^ (red) clones 16 h after olaparib (5 μM), ibrutinib (5 μM) and/or bendamustine (50 μM). Data are shown as the mean values ± SD of at least 50 comets analyzed per condition in three independent experiments.

Taking into account these results, it could be suggested that the mechanism of synergy of olaparib and ibrutinib in del(11q) cells could be explained, at least in part, by synthetic lethality. Therefore, the addition of a DNA damage-inducing agent should increase the cytotoxicity of this combination due to large amounts of unrepaired DSBs. Interestingly, the addition of bendamustine to olaparib and ibrutinib synergistically reduced viability in all the HG3-del(11q) and MEC1 *ATM*^KO^ CRISPR/Cas9-edited clones (Fig. 6a; Supplementary Fig. S8a). In addition, the combination of the three of these drugs also resulted in decreased viability in a subset of stimulated CLL primary cells (Fig. 6b).

To validate that this reduction in cell viability was due to the accumulation of lethal DSBs, neutral comet assays were performed on HG3^WT^ and HG3-del(11q) *ATM*^KO^ cells after treatment with olaparib, ibrutinib and/or bendamustine. Remarkably, the triple combination of olaparib, ibrutinib and bendamustine led to larger amounts of unrepaired DSBs (Fig. 6c, Supplementary Fig. 8b). Of note, the dual combinations of olaparib + ibrutinib or ibrutinib + bendamustine induced higher levels of DNA damage than these drugs in monotherapy, supporting the aforementioned ibrutinib-mediated impairment of HR repair. In addition, HG3-del(11q) *ATM*^KO^ cells exhibited more DNA damage upon olaparib + ibrutinib and the triple combination than HG3^WT^ cells, providing further evidence of the selective hypersensitivity of del(11q)/*ATM*^KO^ CLL cells to PARP inhibitors in combination with ibrutinib.

## Discussion

The advent of next-generation sequencing has made feasible to unveil CLL as a highly genetically heterogeneous disease [12, 13]. Specifically, del(11q) patients represent a heterogeneous group inclusive of individuals carrying bi-allelic inactivation of *ATM* [6, 14, 15]. The absence of cellular models harboring this deletion and the difficulty of collecting large cohorts of patients harboring all the possible combinations of del(11q) and/or *ATM* truncating mutations, have left remaining questions about the biological effects and treatment response related to these genomic alterations. In this study, we explored the implementation of the CRISPR/Cas9 technology to generate in vitro CLL models carrying del(11q) and/or *ATM* mutations. In this fashion, we generated unique isogenic cell lines mimicking the *ATM*-related genomic heterogeneity seen in CLL patients.

Considering the genetic intratumoral heterogeneity underlying CLL patients, we have generated del[11q)/*ATM*-mutated models in two different cytogenetic backgrounds (del(13q) in HG3 and del(17p) in MEC1). In addition, multiple driver mutations can co-occur within the same tumoral clone, usually driving clonal expansion of CLL cells [44, 45]. Therefore, it is important to study how genetic alterations could synergistically act within the same tumor cell. To this extent, our CRISPR/Cas9 model is the first of its kind analyzing the biological impact of concurrent del(11q) and *ATM* mutations.

Our data show that these models faithfully represent the biology of *ATM* loss in the pathogenesis of CLL. In this way, the response of our CRISPR/Cas9-generated isogenic cell models to γ-irradiation was analyzed, showing that biallelic loss of *ATM* strongly impaired γH2AX foci formation after irradiation [46], leading to the accumulation of unrepaired DSBs. Interestingly, del(11q) CLL cells also showed moderate levels of unrepaired DNA damage after irradiation and biallelic *ATM* inactivation lead to higher levels of unrepaired DNA damage. Of note, del(11q) CLL patients displayed a higher rate of genomic alterations than patients without del(11q) [47, 48]. Therefore, our results suggest that the presence of del(11q) together with *ATM* mutations may be able to increase the risk of developing secondary genetic abnormalities in CLL cells, contributing to the appearance of subclonal genomic alterations frequently observed in CLL patients during the disease course and associated with poor outcomes [12, 44, 49].

Ibrutinib-mediated BCR inhibition has transformed the treatment landscape of CLL. Despite its proved benefits, disease progression on ibrutinib is being increasingly reported and ibrutinib resistance has emerged as a therapeutic challenge [20–24]. In addition, complex karyotype, which is associated with del(11q) [50], has been associated with poor outcome in ibrutinib-treated patients [51]. Therefore, novel therapeutic approaches need to be explored in high-risk CLL patients. Some studies have shown the efficacy of PARP inhibitors in *ATM*-deficient cell lines and murine models, respectively, but not in isogenic del(11q) models or large cohorts of genetically-matched CLL samples [52, 53]. The application of PARP inhibition to our CLL models has highlighted the efficacy of this drug in del(11q) cells with biallelic inactivation of *ATM* in vitro*,* in vivo and ex vivo. Remarkably, olaparib was especially effective in CLL cells with complete dysfunctional ATM protein. Since CLL patients harboring biallelic inactivation of *ATM* represent a group with dismal outcome [6, 14, 15], olaparib could be a rational therapeutic alternative for this high-risk subgroup of CLL patients. Therefore, our CRISPR/Cas9-engineered CLL cell lines may be also suitable models to predict drug response of CLL-related genomic alterations.

Our work provides evidence of synergizing effects of PARP and BCR inhibition in isogenic CLL cell lines as well as in primary CLL cells combining olaparib and ibrutinib treatment. Since ibrutinib has been proven to be one of the most optimal therapeutic strategies for fludarabine-relapsed/refractory CLL patients [54], and olaparib is highly effective in del(11q)/*ATM*-mutated CLL cells, the combination of both drugs could be effective for del(11q)-relapsed/refractory CLL patients. Furthermore, our study suggests that the synergy mechanism could be due to a PI3K signaling-dependent off-target effect of ibrutinib on HR repair through downregulation of RAD51, triggering synthetic lethality when combined with PARP inhibitors. Interestingly, combined PI3K and PARP inhibition have also provided more efficient responses in BRCA1-deficient breast cancer cells [55] and dual ATR and BCR inhibition has also been proven to be synergistic in *ATM*-defective CLL cells [56]. Moreover, it has been reported that BCR inhibitors could increase genomic instability in B cells [57] and transcriptomic data also support the evidence of RAD51 downregulation in CLL patients treated with ibrutinib (Fig. 5a) [23]. In fact, since the addition of ibrutinib to bendamustine plus rituximab can significantly improve outcomes of CLL patients [58] and PARP inhibitors have been proven to be highly effective in HR-impaired breast and ovarian cancer patients [59, 60], relapsed/refractory del(11q)/*ATM*-mutated CLL patients may potentially benefit from this combinatorial strategy.

Altogether, our results highlight that the CRISPR/Cas9 system is an applicable technique for the generation of in vitro CLL models mimicking specific genomic alterations frequently observed in CLL patients. By using these models, we have delved into the knowledge on the effects of monoallelic del(11q) or biallelic *ATM* loss on the DNA damage response signaling in CLL. Furthermore, this work demonstrates that PARP inhibition in combination with ibrutinib may be explored as a therapeutic option for del(11q) CLL patients showing *ATM* biallelic inactivation.

## Supplementary information

Supplemental Material
