# Supplementary material for: CRISPR/Cas9-generated models uncover therapeutic vulnerabilities of del(11q) CLL cells to dual BCR and PARP inhibition
Source: Leukemia. 2020 Jan 23;34(6):1599–612. doi: 10.1038/s41375-020-0714-3 (PMC7266745; doi:10.1038/s41375-020-0714-3)
Supplement: Supplementary file 1 — Supplemental Material [file 41375_2020_714_MOESM1_ESM.docx]

**SUPPLEMENTARY INFORMATION**

**CRISPR/Cas9-Generated Models Uncover Therapeutic Vulnerabilities of del(11q) CLL Cells to Dual BCR and PARP inhibition**

Miguel Quijada-Álamo, María Hernández-Sánchez, Verónica Alonso-Pérez, Ana E Rodríguez-Vicente, Ignacio García-Tuñón, Marta Martín-Izquierdo, Jesús María Hernández-Sánchez, Ana B Herrero, José María Bastida, Laura San Segundo, Michaela Gruber, Juan Luis García, Shanye Yin, Elisa ten Hacken, Rocío Benito, José Luis Ordóñez, Catherine J Wu and Jesús María Hernández-Rivas

This section contains:

1. Supplemental methods
2. 4 supplemental tables
3. 8 supplemental figures

**SUPPLEMENTAL METHODS**

**Cell lines and culture conditions**

The human CLL-derived cell lines HG3 and MEC1 were purchased from DMSZ (Deuthche Sammlung von Mikroorganismen and Zellkulturen). HG3 cells were cultured in RPMI 1640 medium (Life Technologies) supplemented with 15% Fetal Bovine Serum (FBS), 1% glutaMAX and 1% penicillin/streptomycin (Life Technologies). MEC1 cells were cultured in Iscove’sMDM medium (Lonza) supplemented with 10% FBS, 1% glutaMAX and 1% penicillin/streptomycin. HEK 293T cells and HS-5 bone marrow stromal cells were obtained from DMSZ and ATCC (American Type Culture Collection), respectively, and used for lentiviral production and primary CLL co-cultures, respectively. They were maintained in DMEM (Life Technologies) supplemented with 10% FBS, 1% glutaMAX and 1% penicillin/streptomycin. All cell lines were incubated at 37ºC in a 5% CO_2_ atmosphere. The presence of mycoplasma was tested frequently in all cell lines with MycoAlert kit (Lonza), only using mycoplasma-free cells in all the experiments carried out.

**Drugs and Reagents**

Olaparib and ibrutinib, were obtained from LC Laboratories, bendamustine and copanlisib were from Selleckchem and B02 was from Sigma. For the *in vitro* experiments, all drugs were resuspended in DMSO (Sigma). In the *in vivo* model, olaparib was prepared in 2-hydroxyl-propyl-B cyclodextrin 10% (Sigma) in phosphate-buffered saline (PBS) plus 10% DMSO.

**Next-Generation Sequencing**

A targeted-capture next-generation sequencing (NGS) strategy was used to analyze the mutational status of *ATM*, *BIRC3*, *NOTCH1*, *TP53*, *SF3B1*, *XPO1*, *MYD88* and *FBXW7*. These genes were included in a custom-panel of 57 genes previously reported as mutated in CLL or involved in the disease pathogenesis (1, 2) following the approach of Agilent SureDesign. Sequence data generated by Illumina NextSeq 500 was analyzed by an in-house bioinformatic pipeline mapping reads to the Reference Human Genome (hg19). Burrows-Wheeler-Aligner (BWA) and Genome Analysis Tool Kit (GATK) were used for variant-calling of single nucleotide variants (SNVs) and short insertions/deletions (InDels) (3). Supplementary Table S2 shows the regions analyzed for this study and the coverage data for each gene. Filters were set to display sequence variants occurring in more than 2% of reads. Variants were then filtered according to the severity of the consequence, to accept variants leading to an amino acid change in the protein sequence (missense, nonsense, frameshift) and those in the splice site. Minor allelic frequencies (MAFs) were consulted in the Exome Variant Server, 1000 Genomes Browser and exome aggregation consortium (ExAC) databases; removing those with a MAF of >1%. Several in silico tools such as Sorting Intolerant From Tolerant (SIFT) and Polymorphism Phenotyping v2 (PolyPhen-2) (HDIV and HVAR scores) were used to predict the functional effects and pathogenicity of the novel variants. All accepted mutations were reviewed individually by Integrative Genomics Viewer (IGV) (Broad Institute, Cambridge, MA, USA) software. Supplementary Table S3 shows the list of mutations in CLL patients.

**Lentiviral production and cell transduction**

The lentiviral constructions were co-transfected in addition to pMD2.G (Addgene #12259) and psPAX2 (Addgene #12260) into HEK 293T using Lipofectamine 2000^®^ (Life Technologies). Supernatant containing the lentiviral particles was collected 48 and 72 hours after transfection and subsequently concentrated using Lenti-X concentrator^®^ (Clontech). For transduction, fresh lentiviruses were used to infect 5 x 10^5^ cells cultured in medium supplemented with 8 μg/mL polybrene and seeded in a 24-well plate.

Lentiviral particles containing plasmids for expression of Cas9 and blasticidine were transduced into each cell line and selected by blasticidine (20 μg/ml) for two weeks (4). Cas9 activity was tested using a previously reported system (5).

pLKO5 vectors carrying the desired sgRNAs were packed into lentiviral particles and transduced in Cas9-expressing HG3 and MEC1 cell lines. 72 hours after transduction, GFP+ and/or RFP+ cells were flow-sorted and single-cell seeded in 96-well plates. Clones were analyzed by Sanger sequencing to investigate the presence of mutations at the cut sites.

**sgRNAs nucleofection**

To generate del(11q) in HG3 cells, pLKO5 vectors targeting both 11q22.1 and 11q23.3 were nucleofected simultaneously using the Cell Line V Nucleofector Kit (Lonza). Clones were analyzed by Sanger sequencing to investigate the presence of the fusion region between 11q22.1 and 11q23.3 and the positive clones were validated by fluorescence *in situ* hybridization (FISH).

**PCR and sequencing of sgRNA target sites**

Genomic DNA (gDNA) was extracted using the QIAampDNA Micro Kit (Qiagen) following the manufacturer’s instructions. PCR was performed using primers flanking the target sites for the sgRNAs (Table S4). For screening the loss-of-function mutations, PCR products were purified using High Pure PCR Product Purification Kit (Roche) and resulting indels at the expected locations were confirmed by Sanger sequencing. The efficiency of the sgRNAs was assessed by Tracking of Indels by Decomposition (TIDE) software (<https://tide-calculator.nki.nl>; Netherlands Cancer Institute) (6). For del(11q), the resultant product was screened by a pair of primers flanking upstream and downstream of the 2 sgRNA cleavage sites for each deletion. In the absence of deletion, this product is too large to be efficiently amplified. Two additional pair of primers flanking each sgRNA target site being one of the primers internal to the sequence to be deleted were used to characterize the non-deleted allele in monoallelic deletion clones.

**Fluorescence in situ hybridization**

Interphase FISH was carried out in primary CLL samples as well as CRISPR/Cas9 generated cell lines using the commercially available probes: 11q22/*ATM,* 12p11.1-q11 (alpha satellite), 13q14 and 17p13/*TP53* (Vysis/Abbott Co, Abbott Park, IL, USA). Dual-color FISH using differently labeled control and test probes was implemented following the methods previously described (7).

**Flow cytometry**

Virally transduced cell lines were single-cell sorted by fluorescence-activated cell sorting (FACS) in a FACS Aria cytometer (BD Biosciences) using two strategies: 1) for 11q deletion, GFP and RFP double positive cells were sorted; 2) for loss-of-function mutated cells, either RFP and/or GFP cells were selected.

In addition, Cas9 activity of HG3-Cas9 and MEC1-Cas9 cell lines transduced with pXPR-011 plasmid was assessed by flow cytometry. Cells were washed twice in PBS and the samples and the data were acquired in an Accuri C6 Flow Cytometer and analyzed using Flowjo software.

**Western blot analysis**

For whole cell-lysates, cells were washed with PBS and lysed in ice-cold lysis buffer (140 mmol/l NaCl, 50 mmol/l EDTA, 10% glycerol, 1% Nonidet P-40, 20 mmol/l TrisHCl pH 7) containing protease inhibitors (cOmplete^™^) (Roche). Protein concentration was measured using the Bradford assay (BioRad). Protein samples were subjected to SDS-PAGE and transferred to a nitrocellulose membrane (GE Healthcare). After blocking, membranes were incubated with anti-human antibodies. The following primary antibodies purchased from Cell Signaling Technologies were used: anti-ATM (#2873, Rabbit), anti-PARP1 (#9542, Rabbit), anti-p-BTK (#5082, Rabbit), anti-BTK (#8547, Rabbit), anti-p-AKT (#9271, Rabbit), anti-AKT (#9272, Rabbit), anti-HMGB1 (#3935, Rabbit), anti-β-actin (#4967, Rabbit), anti-Vinculin (#4650, Rabbit), anti α/β tubulin (#2148, Rabbit) and anti-GAPDH (#5174, Rabbit). Horseradish peroxidase-linked anti-rabbit antibody (#7074, Cell Signaling Technologies) was used as secondary antibody at 1:5,000 dilution. Antibody signal was detected using ECL^™^ Western Blotting Detection Reagents (RPN2209, GE Healthcare).

**Viability assay**

Cell proliferation was assessed using 3-(4,5-dimethylthiazol-2-yl)-2,5-diphenyltetrazolium bromide (MTT) colorimetric assay (Sigma-Aldrich). After drug treatment, cells were incubated for 2h with a 1:10 MTT solution and subsequently added 1:2 SDS-HCl in agitation for 6 hours. Absorbance was read on an Infinite® F500 Tecan plate reader (Tecan) at 570nm. To define drug-drug interactions (in terms of synergism, additivity, or antagonism), the combination index (CI) was calculated according to the Chou-Talalay method (8, 9), using CalcuSyn software Version 2.0 (Biosoft). Synergy levels were divided into: < 0.1 very strong synergism; 0.1-0.90, synergism (ranging from strong synergism to slight synergism), and 0.90-1.10, nearly additive to additive.

**γH2AX and RAD51 immunofluorescence**

Cells were irradiated at a dose of 2 Gy during the exponential phase of cell growth with γ-rays using a Gammacell 1000 Elite irradiator (Cesium137). After 1 hour (**γ**H2AX) or 6 hour (RAD51) incubation at 37ºC, 5 x 10^4^ cells were fixed and permeabilized onto poly-L lysine-coated slides as previously described (10). After blockade, slides were incubated with anti-γH2AX (Millipore) or anti-RAD51 (Calbiochem) antibodies at 1:1,000 dilution for 2 hours. Cy™5 Goat Anti-Mouse or Cy™3 Goat Anti-Rabbit (Jackson Immunoresearch) were used as secondary antibodies (1:1,000; 1 hour). Nuclei were stained with DAPI (4′,6′-diamidine-2-fenilindol), diluted in PBS and incubated for 3 min in agitation at room temperature. Cells were washed twice in PBS and slides were mounted with Vectashield reagent (Vector Laboratories). Images were acquired using a Leica TCS SP5DMI-6000B confocal microscope (Leica) and analyzed with Leica LAS AF (Leica) and ImageJ software (<https://imagej.nih.gov/ij/)>.

**Comet assay**

Cells were irradiated at a dose of 40 Gy during the exponential phase of cell growth with γ-rays using a Gammacell 1000 Elite irradiator (Cesium137). After irradiation, samples were incubated for 3h at 37ºC and processed for neutral comet assay as previously described (11). In the experiments of drug-induced analysis of DSBs, cells were pre-incubated with olaparib (5 μM), ibrutinib (5 μM) and/or bendamustine (50 μM) for 16 hours and subsequently processed for neutral comet assay. In brief, 2 x 10^5^ cells/ml were adjusted in ice-cold PBS and mixed with LMAgarose (Trevigen), at 37ºC, at a ratio of 1:10 (v/v). Right after, 25 μL of this mixture were transferred onto CometSlide slides (Trevigen) and placed at 4ºC for 10-20 minutes. Slides were incubated in N1 lysis solution (11) overnight at 37°C in the dark. After rinsing twice in N2 buffer (11), slides were subjected to electrophoresis in N2 solution for 25 min at 1V/cm at 4ºC. Samples were stained with ethidium bromide and analyzed with a fluorescence microscope (Zeiss Axioplan 2) equipped with a Hamamatsu Orca-EC camera. Images were obtained using Openlab software. CometScore^™^ software was used for tail moment quantification of at least 50 cells per condition.

**Apoptosis analysis**

Apoptosis in response to drug treatment was measured by flow cytometry with annexin V-Dy634 (Immunostep) according to the manufacturer’s instructions. In brief, 3 x 10^5^ cells were seeded in 24-well plates and treated 48 hours with the drug concentration of interest, then they were labeled with annexin V and propidium iodide (PI). Since RFP+ signal was detected on the same channel as PI in our flow-cytometer, only GFP+ clones could be included in these studies.

**Transwell migration assays**

The migration studies were carried out in Transwell™ plates with permeable polycarbonate membrane inserts (Corning) with 6.5 mm diameter and 5 μm pore size. Briefly, 3 x 10^5^ cells were serum starved and incubated with olaparib, ibrutinib or the combination of both drugs at 1.25 μM concentration. After 12 hours, cells were placed on the top of the Transwell™ membrane and migration towards 200 ng/ml CCL19 (300-29B; Peprotech) was performed. The number of migrated cells was subsequently quantified in an Accuri C6 Flow Cytometer.

**Homologous recombination (HR) activity assay**

The HR activity of HG3 cells after ibrutinib treatment was measured using an HR-reporter plasmid previously described (12). The plasmid was digested with the restriction enzyme I-SceI and purified. HG3 cells were pretreated with ibrutinib for 12 hours and subsequently transfected with the HR digested construct (2 μg) together with the plasmid pDsRed-N1 (2 μg) as a control of positive-transfected cells, using the Cell Line V Nucleofector Kit (Lonza). After transfection, cells were incubated with ibrutinib for another 24 hours. Live cells were selected by FSC/SSC gating, and live GFP+ and DsRed+ cells were quantified by flow cytometry. HR efficiency was calculated as the ratio of GFP+ to DsRed+ cells. One million events per sample were analyzed. Efficiency of HR was calculated by dividing the number of GFP+ cells of the totality of positive-transfected DsRed+ cells.

**Xenograft experiments**

To test the olaparib efficacy in an intravenous xenograft model 20 four-to-five-week-old female NSG mice were used. 3 x 10^6^ cells were resuspended in 100 μL of cellular medium and injected into the tail vein of the mice. Mice were injected either with HG3^WT^ cells (*n* = 10) or HG3-del(11q) *ATM*^KO^ cells (*n* = 10). Seven days after cell injection, mice were randomized and received either 100 mg/kg olaparib, or vehicle, 2-hydroxyl-propyl-B cyclodextrin 10% in PBS plus 10% DMSO, via intraperitoneal injection, twice daily during five days a week. Mice were monitored daily during the survival experiment and sacrificed by anesthesia overdose when they presented symptoms of severe disease (lethargy and >20% loss of body weight).

For the analysis of the engraftment in NSG mice, 40 μL of peripheral blood samples were collected from the tail vain. Red blood cells were lysed with erythrocyte lysis buffer, and the remaining cells were then washed twice in PBS. Samples were stained with fluorophore-conjugated antibodies against mouse-CD45 (PerCP-Cy5.5, BD Biosciences), human-CD45 (hCD45) (CF Blue, Immunostep), CD19 (PE-Cy7, Immunostep) and CD5 (APC, BD Biosciences). Data were obtained on a FACSAria flow cytometer and analyzed with FlowJo software.

**Supplemental References**

1. Landau DA, Tausch E, Taylor-Weiner AN, Stewart C, Reiter JG, Bahlo J, et al. Mutations driving CLL and their evolution in progression and relapse. Nature. 2015 Oct 14;526(7574):525–30.

2. Puente XS, Beà S, Valdés-Mas R, Villamor N, Gutiérrez-Abril J, Martín-Subero JI, et al. Non-coding recurrent mutations in chronic lymphocytic leukaemia. Nature. 2015 Oct 22;526(7574):519–24.

3. DePristo MA, Banks E, Poplin R, Garimella K V, Maguire JR, Hartl C, et al. A framework for variation discovery and genotyping using next-generation DNA sequencing data. Nat Genet. 2011 May 10;43(5):491–8.

4. Sanjana NE, Shalem O, Zhang F. Improved vectors and genome-wide libraries for CRISPR screening. Nat Methods. 2014 Aug 1;11(8):783–4.

5. Doench JG, Hartenian E, Graham DB, Tothova Z, Hegde M, Smith I, et al. Rational design of highly active sgRNAs for CRISPR-Cas9–mediated gene inactivation. Nat Biotechnol. 2014 Dec 3;32(12):1262–7.

6. Brinkman EK, Chen T, Amendola M, van Steensel B. Easy quantitative assessment of genome editing by sequence trace decomposition. Nucleic Acids Res. 2014 Dec 16;42(22):e168.

7. González MB, Hernández JM, García JL, Lumbreras E, Castellanos M, Hernández JM, et al. The value of fluorescence in situ hybridization for the detection of 11q in multiple myeloma. Haematologica. 2004 Oct;89(10):1213–8.

8. Chou TC, Talalay P. Quantitative analysis of dose-effect relationships: the combined effects of multiple drugs or enzyme inhibitors. Adv Enzyme Regul. 1984;22:27–55.

9. Chou TC, Motzer RJ, Tong Y, Bosl GJ. Computerized quantitation of synergism and antagonism of taxol, topotecan, and cisplatin against human teratocarcinoma cell growth: a rational approach to clinical protocol design. J Natl Cancer Inst. 1994 Oct 19;86(20):1517–24.

10. Herrero AB, San Miguel J, Gutierrez NC. Deregulation of DNA double-strand break repair in multiple myeloma: implications for genome stability. Ricchetti M, editor. PLoS One. 2015 Mar 19;10(3):e0121581.

11. Olive PL, Banáth JP. The comet assay: a method to measure DNA damage in individual cells. Nat Protoc. 2006 Jun;1(1):23–9.

12. MAO Z, BOZZELLA M, SELUANOV A, GORBUNOVA V. Comparison of nonhomologous end joining and homologous recombination in human cells. DNA Repair (Amst). 2008 Oct 1;7(10):1765–71.

**Supplemental Table 1. Clinical and biological characteristics of CLL patients.**

| **Sample ID** | **Sex** | **Treatment** | **IGHV status** | **Cytogenetics (FISH)** | **% del(11q)** | ***ATM* mutations** | **ATM status** | **Other mutated CLL drivers** | **Sample used in** |
| --- | --- | --- | --- | --- | --- | --- | --- | --- | --- |
| **ID-01** | F | No | NA | del(13q) |  |  | WT |  | Fig.2b, 4 |
| **ID-02** | M | No | UM | del(11q) | 59% |  | Monoallelic inactivation |  | Fig.2b, 4 |
| **ID-03** | M | No | UM | del(11q), del(13q), +12 | 65% | p.R2993X (85%) | Biallelic inactivation | *NOTCH1* | Fig.2b, 4 |
| **ID-04** | F | No | M | del(11q), del(13q) | 27% |  | Monoallelic inactivation |  | Fig.2b, 4 |
| **ID-05** | M | No | UM | del(11q) | 75% | p.C2159X (67%) | Biallelic inactivation |  | Fig.2b, 4 |
| **ID-06** | M | No | UM | del(11q), del(13q) | 95% |  | Monoallelic inactivation | *XPO1* | Fig.2b, 4 |
| **ID-07** | M | Yes | UM | del(11q), del(13q) | 60% | p.G2023R (86%) | Biallelic inactivation | *SF3B1* | Fig.2b, 4 |
| **ID-08** | F | No | NA | Normal |  |  | WT |  | Fig.2b, 4 |
| **ID-09** | M | No | NA | del(11q), del(13q) | 80% | p.R337C (95%) | Biallelic inactivation | *SF3B1* | Fig.2b, 4 |
| **ID-10** | F | No | UM | del(13q) |  |  | WT | *SF3B1, NOTCH1* | Fig.2b, 4 |
| **ID-11** | F | Yes | NA | del(11q), del(13q) | 90% |  | Monoallelic inactivation |  | Fig.2b, 4 |
| **ID-12** | M | Yes | M | del(11q) | 95% |  | Monoallelic inactivation | *SF3B1* | Fig.2b, 4 |
| **ID-13** | M | No | UM | del(13q) |  |  | WT | *NOTCH1, XPO1* | Fig.2b, 4 |
| **ID-14** | M | No | M | del(13q) |  |  | WT | *MYD88* | Fig.2b, 4 |
| **ID-15** | M | No | M | +12 |  |  | WT | *FBXW7* | Fig.2b, 4 |
| **ID-16** | F | No | M | Normal |  |  | WT |  | Fig.2b, 4 |
| **ID-17** | M | No | M | del(13q) |  |  | WT |  | Fig.2b, 4 |
| **ID-18** | M | No | UM | Normal |  | p.I2260fs (6%); p.Y2019C (8%) | Monoallelic inactivation | *SF3B1* | Fig.2b, 4 |
| **ID-19** | F | Yes | M | del(13q) |  |  | WT |  | Fig.4, 5b |
| **ID-20** | M | NA | UM | del(11q), del(13q) | 80% | p.C532Y (18%); p.L1162fs (13%); p.Q2433fs (44%) | Biallelic inactivation |  | Fig.4, 5b, 6b |
| **ID-21** | M | NA | UM | del(11q), del(13q), +12 | 70% |  | Monoallelic inactivation | *BIRC3, XPO1, NOTCH1* | Fig.4, 5b, 6b |
| **ID-22** | F | No | NA | del(11q), del(13q), +12 | NA | p.1281_1282del (13%) | BIallelic inactivation | *XPO1* | Fig.4, 5b |
| **ID-23** | M | NA | UM | trisomy 12 |  |  | WT | *NOTCH1* | Fig.4, 5b |
| **ID-24** | F | Yes | UM | del(13q), del(17p) |  |  | WT | *TP53, NOTCH1* | Fig.4, 5b |
| **ID-25** | F | No | M | trisomy 12 |  |  | WT | *BIRC3, FBXW7* | Fig.4, 5b |
| **ID-26** | F | No | UM | Normal |  |  | WT | *NOTCH1* | Fig.4, 5b |
| **ID-27** | F | No | M | del(13q) |  |  | WT |  | Fig.4, 5b |
| **ID-28** | F | No | UM | del(13q), del(17p), +12 |  |  | WT | *TP53, FBXW7, NOTCH1* | Fig.4, 5b |
| **ID-29** | F | Yes | UM | Normal |  | p.F1463C (45%) | Monoallelic inactivation | *NOTCH1* | Fig.4, 5b |
| **ID-30** | M | No | M | del(17p) |  |  | WT | *TP53* | Fig.4, 5b |
| **ID-31** | M | No | M | del(13q) |  | p.F1463C (39%) | Monoallelic inactivation |  | Fig.4, 5b |
| **ID-32** | F | Yes | UM | Normal |  |  | WT | *TP53, NOTCH1* | Fig.4, 5b |
| **ID-33** | M | NA | UM | del(11q), del(13q) | 97% |  | Monoallelic inactivation |  | Fig.4, 5b, 6b |
| **ID-34** | M | No | UM | del(13q) |  |  | WT | *XPO1* | Fig.4, 5b, 6b |
| **ID-35** | M | NA | NA | del(13q) |  |  | WT |  | Fig.4, 5b, 6b |
| **ID-36** | M | No | UM | del(11q), del(13q) | 45% |  | Monoallelic inactivation | *TP53* | Fig.4, 5b, 6b |
| **ID-37** | M | No | NA | del(11q), del(13q) | 85% | p.H2125R (79%) | Biallelic inactivation | *SF3B1* | Fig.4, 5b, 6b |
| **ID-38** | F | No | M | del(13q) |  |  | WT |  | Fig.4, 5b, 6b |

**Supplemental Table 2. List of genes and regions analyzed in primary CLL samples and the mean read depth obtained by NGS-approach per gene.**

| **Gen** | **Transcript** | **Regions** | **Mean of reads** |
| --- | --- | --- | --- |
| *ATM* | ENST00000278616 | Exons 2-63 | 489 |
| *BIRC3* | ENST00000263464 | Exons 2-9 | 463 |
| *FBXW7* | ENST00000281708 | Exons 7-12 | 1198 |
| *MYD88* | ENST00000396334 | Exons 2-5 | 2051 |
| *NOTCH1* | ENST00000277541 | Exon 34 and 3'UTR | 2341 |
| *SF3B1* | ENST00000335508 | Exons 14-16, 18 | 669 |
| *TP53* | ENST00000269305 | Exons 4-11 | 1402 |
| *XPO1* | ENST00000401558 | Exons 15-16 | 540 |

**Supplemental Table 3. List of mutations detected by NGS in primary CLL samples included in this study.**

**Supplemental Table 4. Oligos designed for each sgRNA and PCR primers of sgRNA target sites.**

| **Target** | **Forward (5’-3’)** | **Reverse (5’-3’)** |
| --- | --- | --- |
| *ATM* exon 10 sgRNA1 | CACCG**GTAAGGCATCGTAACACATA** | AAAC**TATGTGTTACGATGCCTTAC**C |
| *ATM* exon 10 sgRNA2 | CACCG**GACACAATGCAACTTCCGTA** | AAAC**TACGGAAGTTGCATTGTGTC**C |
| 11q22.1 sgRNA-A | CACCG**AGATGACTTCCTGAACAGTG** | AAAC**CACTGTTCAGGAAGTCATCT**C |
| 11q23.3 sgRNA-B | CACCG**TGCGACCGGACTCAGATCCC** | AAAC**GGGATCTGAGTCCGGTCGCA**C |
| Control sgRNA1 | CACCG**ACGGAGGCTAAGCGTCGCAA** | AAAC**TTGCGACGCTTAGCCTCCGT**C |
| *ATM* exon 10 | TCCTGCCAATTTAGGAAGTAGGAC | CTGCAGGCTGACCCAGTAAA |
| 11q22.1 sgRNA cut site | GCTGCCAGCTTCAATTAGGA | ACAATACCTTATGAGACCTGGTGA |
| 11q23.3 sgRNA cut site | CGTTACGCGTTGAGGCATTT | GAGGCTCGAGATGTAAGCGG |

**Supplemental Figure 1**

**Determination of Cas9 activity in HG3-Cas9 and MEC1-Cas9 cell lines. (A)** Representative plots of isogenic CLL cell lines stably expressing Cas9 protein and transduced with pXR011 plasmid, which delivers GFP and the RNA guide (sgRNA) targeting GFP. HG3 and MEC1 parental cell lines transduced with pXR011 highly expressed GFP whereas HG3-Cas9 and MEC1-Cas9 cells showed lower levels of GFP expression, indicating the activity of the Cas9 protein in these cell lines. **(B)** GFP expression quantification of HG3 and MEC1 cell lines transduced with pXR011. Data is shown as mean ± SD of two independent experiments.

**Supplemental Figure 2**

**Generation of isogenic CLL cell lines with *ATM* mutations using the CRISPR/Cas9 system. (A)** Diagram showing the steps for the generation of HG3/MEC1 *ATM*^KO^ clones. Three clones of each cell line were generated and used in the functional studies. **(B)** Western blot analysis of HG3-edited clones showing ATM protein expression.

**Supplemental Figure 3**

**Evaluation of double strand breaks signaling in HG3 and MEC1-edited clones. (A)** Quantification of the number of γH2AX foci per cell 1 hour after irradiation (2 Gy) in HG3 (left panel) and MEC1 (right panel) WT and *ATM*^KO^ clones. Data are represented as the mean values ± SD of 2 independent experiments. At least 75 cells per experiment and clone were counted. **(B)** Representative images of γH2AX foci formation (red) in MEC1 clones. Upper panel shows non-irradiated MEC1 cells and lower panel represents MEC1 clones 1 hour post-irradiation (2 Gy).

**Supplemental Figure 4**

**Olaparib response of CRISPR/Cas9-edited CLL cell lines *in vitro* and *in vivo*. (A)** HG3-del(11q) and HG3-del(11q) *ATM*^KO^ clones were treated with increasing doses of olaparib and cell viability was assessed by MTT after 72 hours. Surviving fraction is expressed relative to untreated (DMSO) controls. Data is summarized as the mean ± SD of three independent experiments. *P* < 0.05 (*) **(B)** MEC1-edited clones were treated with olaparib (40 μM) and cell viability was assessed by MTT at different time points up to 12 days. Proliferation rates are presented as MTT absorbance units, and data are represented as mean ± SD. **(C)** Quantification of hCD45+/GFP+ cell populations in the peripheral blood of HG3^WT^ and HG3-del(11q) *ATM*^KO^ xenografted mice at different time points after engraftment. Data is shown as mean ± SD.

**Supplemental Figure 5**

**Cell viability analysis of the effects of olaparib in combination with ibrutinib in MEC1 cells.** **(A)** HG3-edited clones were treated with olaparib in combination with ibrutinib or bendamustine and cell viability was assessed by MTT assay 72 hours later. Upper graphs are representative of HG3-del(11q) *ATM*^KO^ clones. Surviving fraction is expressed relative to untreated controls and data is presented as the mean ± SD of three independent experiments. Combination indexes (CI) for all the clones are detailed in the lower tables (CI values < 0.9 indicate synergism). **(B)** MEC1 cells were treated with olaparib in combination with ibrutinib at the indicated doses and cell viability was assessed by MTT assay 72 hours later. Surviving fraction is expressed relative to untreated controls and data is presented as the mean ± SD of three independent experiments. The mean combination index is displayed in the lower box (CI values < 0.9 indicate synergism). **(C)** CRISPR/Cas9-generated HG3 clones were treated with increasing concentrations of bendamustine or ibrutinib and cell viability was measured after 72 hour- treatment. Surviving fraction is expressed relative to untreated controls and data is presented as the mean ± SD of three independent experiments. **(D)** Representative plots from Fig. 3c of annexin V/PI stained HG3^WT^ and HG3-del(11q) cell lines 48 hours after 5 μM olaparib and ibrutinib treatment. Necrotic cells are allocated on the PI+/annexin V- quadrant. **(E)** Cytotoxicity studies by annexin V/PI staining of MEC^WT^ cells treated with 12 μM olaparib and 4 μM ibrutinib for 48 hours. Cytotoxicity is measured as the percentage of PI+ and annexin V+ cells. Data is summarized as the mean ± SD of three independent experiments. **(F)** Cells were treated with 1.25 μM olaparib, ibrutinib or the combination of both for 12 hours. Migration values are normalized with respect to the control (DMSO) condition. Data is shown as the mean ± SD of three independent experiments.

**Supplemental Figure 6**

**Effects of the combination of olaparib and ibrutinib in HS-5 bone marrow stromal cells and IGHV unmutated primary CLL cells. (A)** HS-5 cells were treated with olaparib (5 μM), ibrutinib (1 μM) or the combination of both and cell viability was assessed by MTT assay 5 days later. The drug combination did not affect HS-5 cell viability. Surviving fraction is expressed relative to an untreated control and data is presented as the mean ± SD. **(B)** Response to the combination of olaparib and ibrutinib in IGHV unmutated/*ATM*^WT^ primary cells and *ATM* biallelic inactivated primary CLL cells.

**Supplemental Figure 7**

**Evaluation of RAD51 levels after ibrutinib treatment in HG3 and MEC1 cells**. **(A)** HG3^WT^ and HG3-del(11q) *ATM*^KO^ cells were pretreated for 24 hours with 5 μM olaparib and/or ibrutinib and subsequently irradiated (2 Gy). After 6 hours of incubation, cells were lysed for immunoblot analysis. **(B)** MEC1^WT^ cells were pretreated for 24 hours with increasing doses of ibrutinib and subsequently irradiated (2 Gy) and analyzed for foci formation 6 hours later. Data are represented as the mean values ± SD. Cells were scored RAD51+ when 5 or more foci were formed. At least 100 cells per experiment were counted **(C)** HG3^WT^ cells were pretreated for 12 hours with the PI3K inhibitor copanlisib (10 nM) and/or 5 μM olaparib and subsequently irradiated (2 Gy). After 6 hours of incubation, cells were analyzed for RAD51 foci formation. Data are represented as the mean values for each treatment condition ± SD. Cells were scored RAD51+ when 5 or more foci were formed. At least 100 cells per experiment were scored for quantification. **(D)** HG3^WT^ and HG3-del(11q) *ATM*^KO^ cells were treated with olaparib in combination with the PI3K inhibitor copanlisib (left) or the RAD51 inhibitor B02 (right) and cell viability was assessed by MTT assay 72 hours later. RAD51 and PI3K inhibition potentiated the effects of olaparib treatment in both cell lines. Surviving fraction is expressed relative to untreated controls and data is presented as the mean ± SD of three independent experiments.

**Supplemental Figure 8**

**Effects of the combination of olaparib, ibrutinib and bendamustine in cell viability and DSB accumulation of CRISPR/Cas9-edited clones. (A)** MEC1-edited clones were treated with olaparib, ibrutinib and/or bendamustine and cell viability was assessed by MTT assay 72 hours later. Surviving fraction is expressed relative to untreated controls and data is presented as the mean ± SD of two independent experiments. **(B)** Representative images of comet assays from Fig. 6c.
